# Changes in Retinal Function and Morphology Are Early Clinical Signs of Disease in Cattle with Bovine Spongiform Encephalopathy

**DOI:** 10.1371/journal.pone.0119431

**Published:** 2015-03-10

**Authors:** M. Heather West Greenlee, Jodi D. Smith, Ekundayo M. Platt, Jessica R. Juarez, Leo L. Timms, Justin J. Greenlee

**Affiliations:** 1 Department of Biomedical Sciences and Interdepartmental Toxicology Program, Iowa State University, Ames, IA 50010, United States of America; 2 Virus and Prion Research Unit, National Animal Disease Center, Ames, IA 50010, United States of America; 3 Department of Genetics and Cell Biology and Interdepartmental Toxicology Program, Iowa State University, Ames, IA 50010, United States of America; 4 Department of Animal Science, Iowa State University, Ames, IA 50010, United States of America; Creighton University, UNITED STATES

## Abstract

Bovine spongiform encephalopathy (BSE) belongs to a group of fatal, transmissible protein misfolding diseases known as transmissible spongiform encephalopathies (TSEs). All TSEs are caused by accumulation of misfolded prion protein (PrP^Sc^) throughout the central nervous system (CNS), which results in neuronal loss and ultimately death. Like other protein misfolding diseases including Parkinson’s disease and Alzheimer’s disease, TSEs are generally not diagnosed until the onset of disease after the appearance of unequivocal clinical signs. As such, identification of the earliest clinical signs of disease may facilitate diagnosis. The retina is the most accessible part of the central nervous system, and retinal pathology in TSE affected animals has been previously reported. Here we describe antemortem changes in retinal function and morphology that are detectable in BSE inoculated animals several months (up to 11 months) prior to the appearance of any other signs of clinical disease. We also demonstrate that differences in the severity of these clinical signs reflect the amount of PrP^Sc^ accumulation in the retina and the resulting inflammatory response of the tissue. These results are the earliest reported clinical signs associated with TSE infection and provide a basis for understanding the pathology and evaluating therapeutic interventions.

## Introduction

Bovine spongiform encephalopathy (BSE) belongs to a group of fatal, protein misfolding neurodegenerative disorders called transmissible spongiform encephalopathies (TSEs), all of which are the result of CNS accumulation of proteinase resistant prion protein (PrP^Sc^). Other TSEs include scrapie in sheep, chronic wasting disease (CWD) in cervids and Creutzfeldt-Jakob disease (CJD) in humans. TSEs differ from other protein misfolding neurodegenerative diseases including Parkinson’s disease and Alzheimer’s disease in that they are transmissible from one individual to another [1]. Interspecies transmission of TSEs remains an active area of research [2–5], and may demonstrate that other TSEs could transmit to humans, but transmission of BSE to humans as variant CJD (vCJD) (reviewed in [6]) has resulted in the death of over 200 individuals worldwide.

There is a lack of antemortem assessments and objective measures of TSE disease progression. Though there has been significant effort to detect prions in diagnostic samples (e.g. CSF, blood, urine, saliva, nasal brushings; [7–16]), there are currently no functional or morphological objective measures that could be applied to monitor disease progression or evaluate the effectiveness of intervention strategies.

We and others have demonstrated that PrP^Sc^ accumulates in the retinas of animals infected with TSEs [1,17–27]. The retina is the most accessible part of the CNS and is amenable to non-invasive assessment of morphology and function. We have previously demonstrated that functional changes develop in the retina of cattle inoculated with transmissible mink encephalopathy, a TSE with microscopic features similar to those of BSE [28], months prior to other clinical signs of disease [24]. Here we describe changes in retinal morphology and function over time in animals inoculated with two different strains of BSE; classical foodborne BSE, which transmits to humans as vCJD [6,29,30], and atypical high-type BSE (BSE-H) diagnosed in the US in 2004 [31]. We demonstrate that antemortem assessment of retinal function and morphology identifies changes 12 months post-inoculation (PI) with both classical BSE and BSE-H, which is an average of 11 and 5 months, respectively, before the onset of unequivocal clinical signs in these animals. Further, we demonstrate strain-specific differences in retinal function, accumulation of PrP^Sc^ in the retina, and the retinal glial response to disease. These results suggest that the retina has significant potential for the development of antemortem assessments for prion disease and is an excellent experimental model to investigate the relationship between PrP^Sc^ accumulation, neural function, and neuropathology.

## Materials and Methods

### Ethics Statement

This experiment was carried out in accordance with the Guide for the Care and Use of Laboratory Animals (Institute of Laboratory Animal Resources, National Academy of Sciences, Washington, DC) and the Guide for the Care and Use of Agricultural Animals in Research and Teaching (Federation of Animal Science Societies, Champaign, IL). The protocol was approved by the Institutional Animal Care and Use Committee at the National Animal Disease Center (protocol number: 3985) and Iowa State University (protocol number: 7154).

### Animals and Inoculum

Two different inocula of bovine spongiform encephalopathy (BSE) were compared in this study. Adult Holstein steers (3.8–4.5 years old) were inoculated intracranially with 1 ml of a 10% (wt./vol) brain homogenate as previously described [32]. Twelve animals were inoculated with classical BSE and nine animals were inoculated with BSE-H. The classical BSE inoculum was from a case diagnosed in the U.S. in 2003 and the high-type BSE (BSE-H) inoculum a case diagnosed in the US in 2004 [31]. The *Prnp* sequence from both source animals was consistent with previously reported cattle sequences [31]. The *Prnp* gene of all inoculated animals was sequenced. There were no polymorphisms in the coding region of experimental animals that resulted in an amino acid sequence different from the normal cattle sequence. There are two non-coding polymorphisms in cattle that are associated with PrP^C^ expression levels [33,34]. A 23-bp deletion within the promoter region removes a binding site for the PR58 repressor protein, the second is a 12-bp deletion in intron 1 that removes an SP1 transcription factor-binding site [35]. The presence or absence of these polymorphisms is indicated in in supplementary data (S1 Table). Due to technical, logistical and safety limitations, we were only able to collect high quality antemortem data from a subset of these animals. The inoculated animals were observed daily by animal care staff at NADC, and examined regularly by investigators to determine the onset of clinical disease. Animals were euthanized at the onset of unequivocal signs of clinical disease, but were not allowed to develop severe, end-stage disease. Upon euthanasia, all animals were confirmed positive by western blot or enzyme immuoassay as directed (IDEXX HerdChek BSE-Scrapie Antigen ELISA test kit, Westbrook, ME). Control animals for histopathology included one mock-inoculated animal from this study, one mock-inoculated animal from a TME to cattle transmission study [36] and three mock-inoculated animals from a CWD to cattle transmission study [37]. Control animals for OCT were 8 age-matched Holstein cows housed at an offsite dairy.

**Electroretinography**. Electroretinography was performed prior to inoculation and at 3-month intervals until the animals were euthanized due to the development of clinical disease. Data from 11 animals inoculated with classical BSE and 6 animals inoculated with BSE-H was used for this analysis. An EPIC 4000 visual electrodiagnostic testing system (LKC Technologies, Gaithersburg, MD) with a CMGS-1 Color Mini-Ganzfeld Stimulator (LKC Technologies, Gaithersburg, MD) was used to capture electroretinograms (ERG). The left eye was tested at each time point. The animals were dark adapted for 20 minutes, followed by two scotopic recordings (single white flash 0.024 cd●s/m^2^, single white flash 2.45 cd●s/m^2^).

**Optical Coherence Tomography**. Retinal thickness was measured *in vivo* using optical coherence tomography (OCT). Data from four animals inoculated with classical BSE and five animals inoculated with BSE-H was used for this analysis. A Bioptigen SD-OCT (Bioptigen, Durham, NC USA) was used to capture linear B scans (6 mm; 1000 A scans/B scan). Scans were taken from dorsocentral retina. At each time point at least 10 measurements/animal of retinal thickness were taken from multiple scan frames (using on-screen calipers) to determine an average thickness measurement for each animal.

**Statistics**. Prism 6 for Mac (Graph Pad Software) was used for all statistical analysis. Group differences were analyzed using a non-parametric Mann-Whitney U test.

**Western Blot**. Approximately 0.5 grams brainstem material was analyzed using the Prionics-Check Western Kit as suggested by the manufacturer with minor modifications as described previously [18]. Samples were homogenized at room temperature with homogenization buffer (10% w/v in Prionics buffer) and digested with proteinase K (PK) for 40 min at 48°C. PK-digestion was stopped according to the manufacturer’s protocol and 1 mg of homogenate was loaded onto pre-cast sodium dodecyl sulfate (SDS)-12% polyacrylamide gel electrophoresis (PAGE) gels. SDS-PAGE was performed as described by the manufacturer and the proteins transferred from the gel to a PVDF membrane with transfer buffer. The membranes were blocked with PVDF blocking buffer and either incubated with monoclonal antibody 6H4 (1:10,000 dilution; Prionics AG, Switzerland:) or monoclonal antibody P4 (1:10,000 dilution or 0.1 μg/ml; R-Biopharm AG, Darmstadt, Germany) for 1 hr at room temperature or overnight at 4°C. A biotinylated sheep anti-mouse secondary antibody (GE Healthcare, Buckinghamshire,UK) at 0.05 μg/mL and a streptavidin-horseradish peroxidase (HRP) conjugate (GE Healthcare, Buckinghamshire, UK) were used according to the manufacturer’s instructions in conjunction with a chemifluorescent detection system (ECL Plus detection system, GE Healthcare, Buckinghamshire, UK) and imaged using a multimode scanner.

**Immunohistochemistry**. Paraffin-embedded tissues were analyzed using immunohistochemistry. Slides were stained by an automated immunohistochemical method for detection of PrP^Sc^ as described previously [32] with slight modifications. Briefly, after deparaffinization and rehydration, tissue sections were autoclaved for 30 minutes in an antigen retrieval solution (DAKO Target Retrieval Solution, DAKO Corp., Carpinteria, CA) and stained with an indirect, biotin-free staining system containing an alkaline phosphatase labeled secondary antibody (*ultra*view Universal Alkaline Phosphatase Red Detection Kit, Ventana Medical Systems, Inc., Tucson, AZ) designed for an automated immunostainer (NexES IHC module, Ventana Medical Systems, Inc., Tucson, AZ). The primary antibodies used were F99/97.6.1 (O’Rourke; Pullman, Washington) at a concentration of 5 μg/ml, and 12B2 [38] at a concentration of 0.2 μg/ml and were incubated at 37°C for 32 minutes. Slides were counterstained with Gill’s hematoxylin and bluing agent (Ventana Medical Systems, Tucson, AZ) then coverslipped. Immunoreactivity for all other antibodies was visualized with an EnVision HRP-System as directed (DAKO). Rabbit anti-GFAP (DAKO) was diluted at 1:7500; rabbit anti Iba-1 (WAKO Chemicals; Richmond, VA) was diluted 1:500, and rabbit-anti-PKC-alpha (SIGMA Chemical; St. Louis, MO) was diluted 1:7500. Images for the figures were captured using a Nikon DS camera on a Nikon Eclipse 80*i* microscope.

## Results

**Fig 1:**
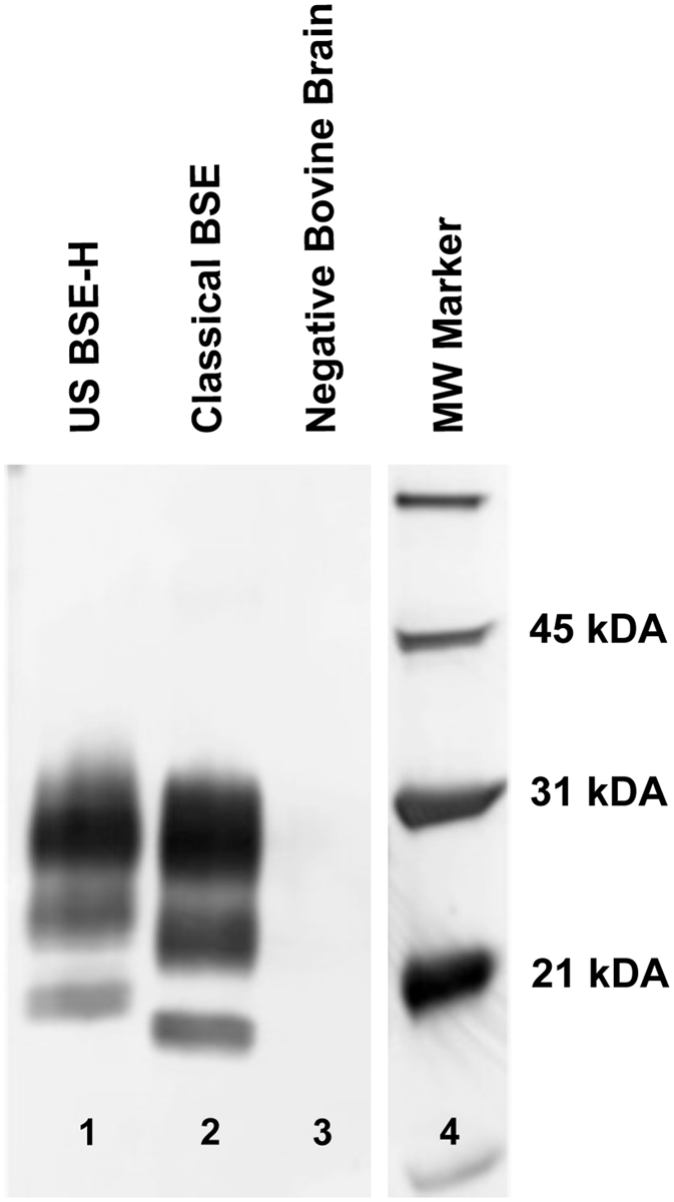
Western blot migration patterns of BSE-H and classical BSE. Immunoblotting for PrP^Sc^ reveals the three characteristic glycoforms. A proteinase K-digested brain homogenate sample from an animal inoculated with H-Type BSE (lane 1) compared to a brain sample from an animal inoculated with classical BSE (lane 2) illustrates the higher molecular weight most noticeable in the BSE- H unglycosylated band. In a brain sample from a negative control animal (lane 3), the proteinase K pre-treatment destroys all antigenicity. Lane 4 contains molecular weight markers.

Incubation time for classical BSE and BSE-H is reported here as the time from inoculation to the time when unequivocal signs of clinical disease were present. Clinical signs of disease included abnormalities in gait or stance, moderate to severe ataxia, and hyperreaction to stimuli such as noise or movement. The average incubation time to onset of unequivocal clinical signs for cattle inoculated with BSE-H was 17.1 months (+/- 0.2 months), the average incubation time for cattle inoculated with classical BSE was 22.8 months (+/- 1.5 months). These differences in incubation time were highly statistically significant (p<0.0001). Upon euthanasia, the migration pattern of PrP^Sc^ on western blots from brainstem homogenates was analyzed to confirm that animals inoculated with classical BSE or US BSE-H retained a classical or high-type migration pattern respectively (Fig. 1).

### Changes in retinal function are detectable during disease incubation

Electroretinography measures the retina’s response to flashes of light at various intensities. We used electroretinography to assess retinal function over the course of incubation with classical BSE and BSE-H. Baseline values were established from ERGs collected prior to inoculation. Cattle were assessed with both a low intensity flash of light (-20 dB, or 0.024 cd●s/m^2^) and a high intensity flash of light (0 dB, 2.45 cd●s/m^2^). The b-waves of the resulting electroretinograms were analyzed (Tables 1 and 2). In animals inoculated with classical BSE, there was not any notable change in the b-wave amplitudes under any testing condition. In animals inoculated with BSE-H there was a decrease in the b-wave amplitude of clinically ill animals (Tables 1 and2), though this decrease did not reach statistical significance. There were, however, significant changes in the b-wave implicit times of animals inoculated with both classical BSE and BSE-H. In clinically ill animals (within several days of necropsy), the b-wave implicit times were significantly prolonged when compared to the animals’ baseline values (Tables 1 and 2). To ensure that the prolongation of the b-wave was not due simply to aging, we compared the animals’ baseline values, collected when they were approximately 4 years of age, to b-wave implicit time values collected when they were approximately one year of age. At 4 years of age, the average baseline implicit time for-20 dB was approximately 54 milliseconds and 27 milliseconds for 0dB. An ERG was also recorded from these same animals at approximately one year of age (3 years prior to their inoculation with BSE). At this time the b-wave implicit time for-20 dB was approximately 58 milliseconds, and 29 milliseconds at 0dB, demonstrating virtually no change in the b-wave implicit time for these animals over three years time. Further, we compared the baseline results to previously published ERG values from Holstein steers at approximately 2 years of age [24]. In the non-inoculated animals in that study (at 2 years of age), the average b-wave implicit time for-20 dB was 62 milliseconds, and 29 milliseconds for 0dB. So, when our comparing 4-year old animals with a different population of 2-year old animals, we do not see any difference in the implicit time of the b-wave. To determine the time during the disease course when a prolonged b-wave implicit time was first detectable, we analyzed ERGs collected from animals at 9, 12 and 15 months post inoculation (MPI). For both classical BSE and BSE-H, b-wave implicit time values first became significantly prolonged at 12 MPI (Tables 1 and 2).

**Table 1:** Changes in the electroretinograms over time in animals inoculated with classical BSE.

Classical BSE	-20 dB Amplitude (μV)	0dB Amplitude (μV)	-20 dB Implicit Time (milliseconds)	0 dB Implicit Time (milliseconds)
Baseline ***11***	276.6 (26.4)	693.5 (48.2)	54.9 (2.0)	28.5 (1.3)
9 MPI ***11***	295.6 (20.7)	790.3 (43.5)	56.2 (2.1)	30.3 (2.4)
12 MPI ***11***	366.5 (28.1)	821.0 (46.5)	63.2** (2.0)	35.2* (2.0)
15 MPI ***11***	279.4 (20.7)	691.3 (35.9)	61.3* (3.7)	33.0 (2.5)
Clinical ***4*** (22.8 MPI)	310.3 (15.1)	796.3 (63.7)	65.0* (2.4)	35.8* (1.9)

B-wave amplitude and implicit time in dark-adapted animals with either dim (-20 dB) or bright (0 dB) flash were analyzed over time. Sample size for each time point are in italics. Values in parenthesis the standard error of the mean. MPI = months post inculcation.

* = p < 0.05;

** = p< 0.01.

**Table 2:** Changes in the electroretinograms over time in animals inoculated with US BSE-H.

BSE-H	-20 dB Amplitude (μV)	0dB Amplitude (μV)	-20 dB Implicit Time (milliseconds)	0 dB Implicit Time (milliseconds)
Baseline ***9***	234.4 (27.8)	672.4 (39.7)	52.7 (1.6)	25.4 (0.8)
9 MPI ***9***	250.2 (28.0)	599.4 (58.7)	55.4 (2.3)	29.7(3.5)
12 MPI ***6***	369.2 (35.6)	815.4 (93.1)	71.3*** (3.7)	41.7* (3.5)
15 MPI ***6***	216.2 (17.5)	509.9 (38.4)	71.7** (5.4)	48.4***(6.4)
Clinical ***3*** (17.1 MPI)	118.4 (54.2)	459.3 (109.6)	89.0** (0.8)	76.2** (3.4)

B-wave amplitude and implicit time in dark-adapted animals with either dim (-20 dB) or bright (0 dB) flash were analyzed over time. Sample size for each time point is in italics. Values in parenthesis are the standard error of the mean. MPI = months post inculcation.

* = p < 0.05;

** = p< 0.01;

*** = p< 0.0001.

### Changes in retinal morphology are detectable during disease incubation

Optical coherence tomography (OCT) was used to measure retinal thickness in dorsocentral over the course of incubation with classical BSE and BSE-H. Baseline values were established from OCT images taken prior to inoculation. The average baseline value for all experimental animals was 300 μM (+/- 8.3). Retinal thickness measurements from a separate group control animals included an age-matched mock-inoculated steer with a retinal thickness measurement of 307 μM, and eight age-matched Holstein cows from an offsite dairy with an average measurement of 293 μM (+/- 5.2). We analyzed OCT images collected approximately 6 months, 12 months and 15 months post inoculation. Retinal thickness values from animals inoculated with BSE-H at, 12 and 15 months post inoculation were markedly, but not significantly, decreased when compared to baseline values (Table 3). However, at 12 and 15 months post inoculation, retinal thickness values were significantly decreased in animals inoculated with classical BSE (Table 3).

**Table 3:** Changes in retinal thickness over time in animals inoculated with classical BSE and US BSE-H.

	Average Retinal Thickness (μM) Classical BSE	Average Retinal Thickness (μM) BSE-H
Baseline	288.4 ***5*** (10.1)	311.6 ***5*** (11.8)
6 MPI	ND	296.0 ***2*** (35.0)
12 MPI (compared to baseline)	243.8* ***4*** (5.0)	272.8 ***5*** (14.7)
15 MPI (compared to baseline)	240.5* ***3*** (1.4)	264.0 ***3*** (5.2)
	Average Retinal Thickness (μM)	
Age Matched Control Herd ***8***	293.1 (5.2)	
Pooled 12 MPI ***9*** (compared to control herd)	259.9***	

Total retinal thickness was measured over time using optical coherence tomography. Animals were compared to their baseline values in 6, 12 and 15 months post inoculation (MPI), and compared to the control cohort at 12 MPI. Values in parenthesis the standard error of the mean, values in itallics in are the sample size, significant changes are indicated by shading.

* = p < 0.05;

** = p< 0.01;

*** = p< 0.0001.

### Sensitivity and specificity of retinal assessment to detect BSE-inoculated cattle at 12 months post inoculation

We calculated the 95% CI of the mean values of b-wave implicit time and retinal thickness from animals at 12 months post inoculation (all values from inoculated animals were pooled), and applied these criteria to baseline values (negative animals) and individual 12 months post-inoculation values (known positive animals) to estimate the specificity and sensitivity of our assays respectively. Individual animal values are provided in supplementary data (S1 Table). In this analysis, b-wave implicit time at-20 dB was 94.7% specific (1/19 animals at baseline would have been a false positive) and 94.7% sensitive (18/19 animals at 12 MPI were above the ‘positive’ criteria). B-wave implicit time at 0dB was 90% specific and 94.1% sensitive. Retinal thickness measured by OCT was 77.7% specific (4/18 negative animals would have been called positive) and 88.9% sensitive (8/9 animals at 12 MPI were thinner than the maximum ‘positive’ value). It is notable, that when analyzed as a group, retinas from animals inoculated with BSE-H were not significantly thinner (Table 3), however, all but one BSE-H inoculated cattle would have been detected when our criteria were applied to each individual animal’s retinal thickness measurement.

### HE examination of retina

**Fig 2:**
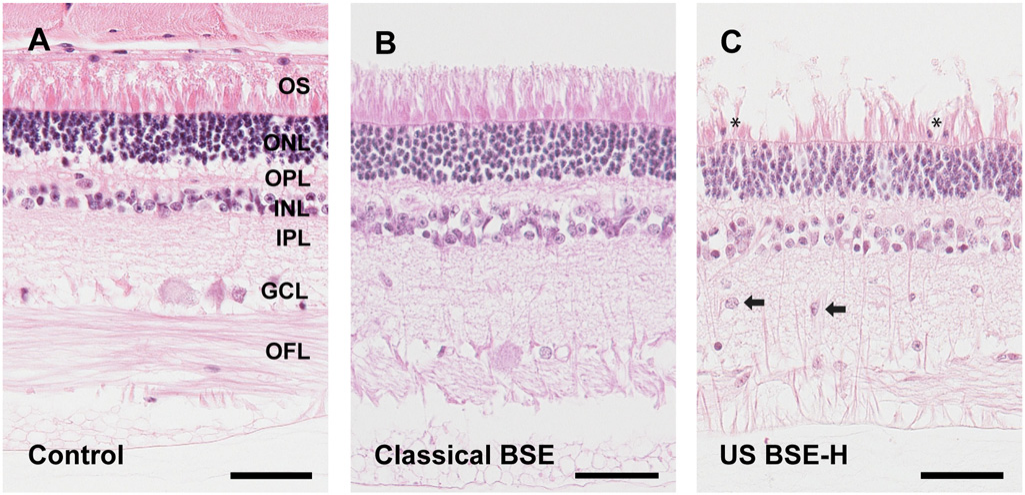
Subtle microscopic changes in retinas from BSE-H inoculated animals. There were no consistent differences between retinal sections from sham inoculated cattle and cattle inoculated with classical BSE (Fig. 2A and 2B). However, sections from animals inoculated with BSE-H (Fig. 2C) had increased numbers of nuclei present amongst outer segments (asterisks) that were morphologically consistent with those of the outer nuclear layer and had increased numbers of large cells from the inner nuclear layer intermingled in the inner plexiform layer (arrows). The ages of the animals in the figure are 4.9, 5.9 and 5.5 years in A, B and C respectively. Abbreviations: OFL = optic fiber layer, GCL = ganglion cell layer; IPL = inner plexiform layer; INL = inner nuclear layer; OPL = outer plexiform layer; ONL = outer nuclear layer; OS = outer segments. Hematoxylin and Eosin. Scale bars = 50 μM.

Subtle differences were noted between the retinas from cattle inoculated with BSE-H as compared to those inoculated with classical BSE or sham inoculated controls. BSE-H inoculated animals had increased numbers of nuclei morphologically consistent with those of the outer nuclear layer present amongst outer segments and had increased numbers of large cells from the inner nuclear layer intermingled in the inner plexiform layer (Fig. 2). In addition, there was a mild increase in cells with small, oblong intensely basophilic nuclei within the inner plexiform layer.

### PrP^Sc^ accumulation in the retina differs between BSE-H and classical BSE

**Fig 3:**
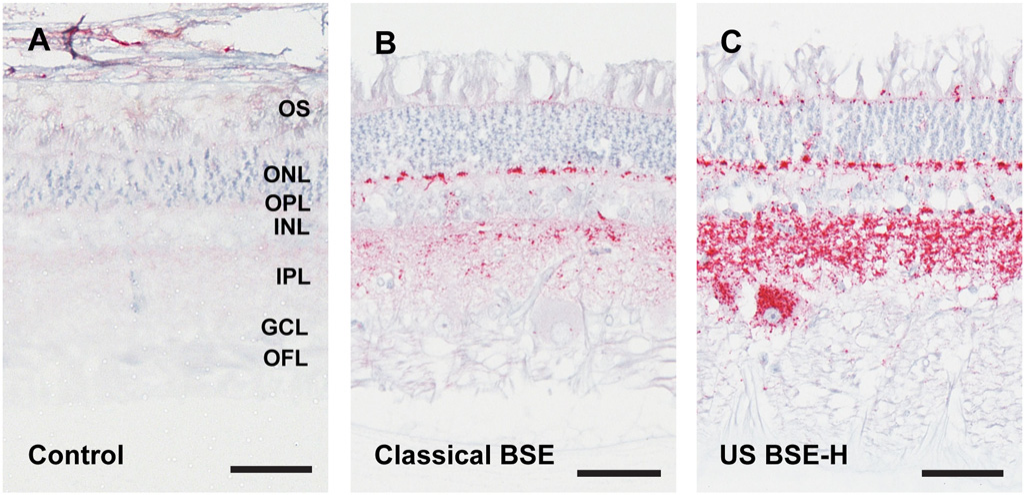
Accumulation of PrP^Sc^ in retinas of cattle inoculated with BSE. Sham inoculated and negative control animals had no PrP^Sc^ immunoreactivity (Fig. 3A). The immunoreactivity in cattle inoculated with classical BSE was limited to the outer and inner plexiform (synaptic) layers (Fig. 3B). BSE-H inoculated cattle (Fig. 3C) had relatively more intense immunoreactivity in the plexiform layers, the outer limiting membrane at the base of photoreceptor outer segments, and large retinal ganglion cells. The ages of the animals in the figure are 6.8, 5.9 and 5.5 years in A, B and C respectively. Abbreviations: OFL = optic fiber layer, GCL = ganglion cell layer; IPL = inner plexiform layer; INL = inner nuclear layer; OPL = outer plexiform layer; ONL = outer nuclear layer; OS = outer segments. Monoclonal antibody 99/97.6.1. Scale bars = 50 μM.

Accumulation of PrP^Sc^ in the retina was assessed with immunohistochemistry (Fig. 3). All prion infected cattle in this study had PrP^Sc^ immunoreactivity in the retina, however, the pattern of deposition was different depending on whether the inoculum source was classical BSE or BSE-H. In animals inoculated with classical BSE, PrP^Sc^ accumulated primarily as multifocal to coalescing granular to globular deposits in the inner and outer plexiform (synaptic) layers (Fig. 3B) with retinal ganglion cells only rarely affected. PrP^Sc^ accumulation was more extensive in animals inoculated with BSE-H: both increased in amount and present within more layers of the retina relative to cattle inoculated with classical BSE. PrP^Sc^-immunoreactivity in inner and outer plexiform layers was intense and formed uniform, intense bands of immunoreactive deposits that extended along the entire length of the retinal sections examined (Fig. 3C). The ganglion cells throughout the retinas of BSE-H inoculated cattle contained large globular deposits immunoreactive for PrP^Sc^. In addition, retinas from BSE-H inoculated cattle contained multifocal globular deposits immunoreactive for PrP^Sc^ between cells of the inner and outer nuclear layers and less frequently amongst outer segments. In addition we examined immunoreactivity for PrP^Sc^ using the 12B2 antibody [38], whose epitope is present in PrP^Sc^ in animals with H-type BSE, but not classical BSE. With this antibody, retinal sections from animals inoculated with classical BSE were indistinguishable from controls, while all retinal sections from BSE-H inoculated animals had 12B2 immunoreactivity in the inner and outer plexiform layers as well as ganglion cells (data not shown). PrP^Sc^-immunoreactivity using either antibody was sufficient for blinded investigators to sort slides of classical BSE from BSE-H with 100% accuracy.

### Disease associated pathology of retinal cells

**Fig 4:**
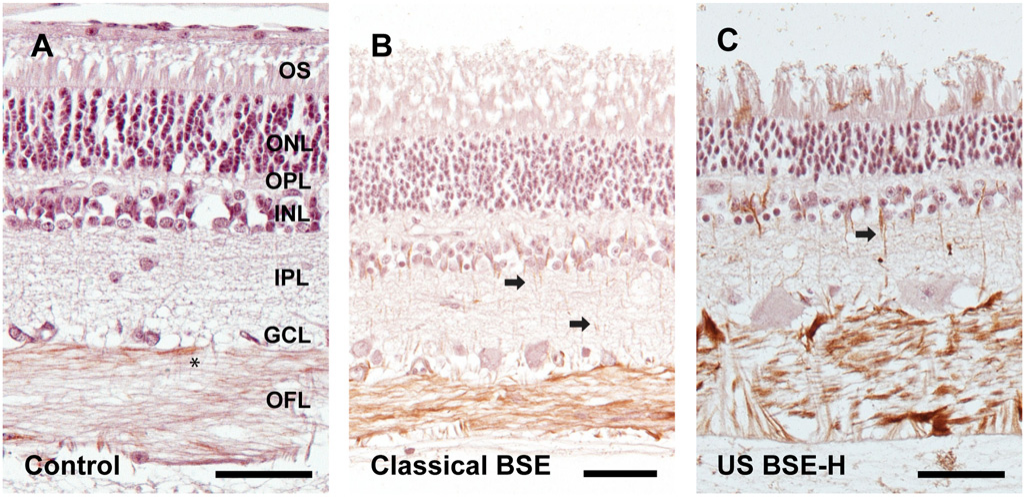
Müller glia are activated in the retinas of cattle inoculated with BSE. An antibody directed against glial fibrillary acidic protein (GFAP) was used to label Müller glia in retinal sections. Retinas from sham inoculated and negative control cattle had GFAP-immunoreactivity only in the optic fiber layer (asterisk), in astrocytes, and endfeet of Müller glia (Fig. 4A). Retinal sections from cattle inoculated with classical BSE had increased immunoreactivity in the optic fiber layer and in occasional thin processes of Müller glia spanning the inner plexiform layer (arrows; Fig. 4B). Sections from cattle inoculated with BSE-H (Fig. 4C) had robust immunoreactivity in the optic fiber layer and consistently in Müller glial processes (arrow), which appeared hypertrophied compared to sections from animals inoculated with classical BSE. The ages of the animals in the figure are 2.7, 6.4 and 5.7 years in A, B and C respectively. Abbreviations: OFL = optic fiber layer, GCL = ganglion cell layer; IPL = inner plexiform layer; INL = inner nuclear layer; OPL = outer plexiform layer; ONL = outer nuclear layer; OS = outer segments. Scale bars = 50 μM.

The b-wave of the electroretinogram is generated by two retinal cell types; bipolar cells and Müller glia [39]. We used immunohistochemistry to assess any potential differences in these cell types in retinas from BSE-inoculated animals. Immunoreactivity for the alpha subunit of protein kinase-C (PKC-alpha) was used to examine the morphology of rod bipolar cells [24]. While we observed some subtle differences between samples, such as what appeared to be an increase of PKC-alpha immunoreactive processes in the retinas of animals inoculated with classical BSE, these differences were not sufficient for blinded investigators to sort inoculated samples from control samples (data not shown). Müller glia are the endogenous glial cells of the retina that express glial fibrillary acidic protein (GFAP). In the normal, healthy retina, GFAP is localized to their endfeet in the retina’s inner limiting membrane. Retinal stress causes the Müller glia to upregulate GFAP, which becomes localized throughout the cell. GFAP-immunoreactivity in the control animals was localized to the Müller glia endfeet and astrocytes in the optic fiber layer (Fig. 4A). In the retinas of animals inoculated with classical BSE, there is a slight increase in GFAP-immunoreactivity in Müller glia endfeet, and the GFAP-immunoreactivity occasionally extended to portions of the cells spanning other layers of the retina (Fig. 4B). In contrast, GFAP-immunoreactivity in the retinas of animals inoculated with BSE-H was intense and extended from the Müller glia endfeet in the inner retina, to their apical processes in the retina’s outer limiting membrane (Fig. 4C). These differences in GFAP-immunoreactivity were sufficient for blinded investigators to sort slides of classical BSE from BSE-H with 100% accuracy.

**Fig 5:**
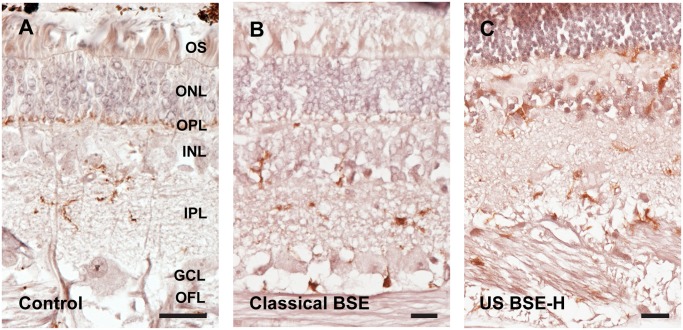
Activation of microglia in retinas of cattle inoculated with BSE. An antibody directed against Iba-1 was used to label microglia in retinal sections. Microglia in retinal sections from sham inoculated and negative control cattle had a stratified appearance with processes primarily in the outer plexiform and inner plexiform layers (Fig. 5A). In sections from cattle inoculated with classical BSE, the microglia appeared to be more amoeboid in morphology (Fig. 5B) but not more numerous than was observed in negative controls. In contrast, retinal sections from cattle inoculated with BSE-H had more numerous microglia with an amoeboid morphology (Fig. 5C). The ages of the animals in the figure are 6.8, 5.9 and 5.7 years in A, B and C respectively. Abbreviations: OFL = optic fiber layer, GCL = ganglion cell layer; IPL = inner plexiform layer; INL = inner nuclear layer; OPL = outer plexiform layer; ONL = outer nuclear layer; OS = outer segments. Scale bars = 20uM

Iba-1-immunoreactivity was used to examine the morphology and prevalence of microglia in the retinas of animals infected with classical BSE and BSE-H. In sham-inoculated control animals, Iba-1-immunoreactivy showed ramified microglia with many visible processes in the inner and outer plexiform layers (Fig. 5A). In retinas from animals inoculated with classical BSE, Iba-1-immunoreactivity showed microglia with a more ameboid-like morphology characteristic of activated microglia (5B). In retinas from animals inoculated with BSE-H, Iba-1-immunoreactivity showed more numerous ameboid-like microglia than were observed in animals inoculated with classical BSE (Fig. 5C). These differences in Iba-1-immunoreactivity were sufficient for blinded investigators to sort slides of classical BSE from BSE-H with 100% accuracy.

## Discussion

Based on our observations reported here, changes in retinal function and morphology are the earliest clinical signs of BSE infection described to date. Optical coherence tomography (OCT) is a non-invasive imaging method that is widely used in human ophthalmology to generate a cross-sectional image of the retina *in vivo*. Here we employed OCT to determine if there was any change in retinal thickness associated with BSE infection over time. We previously reported retinal thinning detectable using OCT in an animal at terminal stages of BSE infection [18], and here we demonstrate that retinal thinning is detectable 12 months post inoculation (MPI) in cattle inoculated with either classical BSE or BSE-H. Our results can be directly related to a careful study of the clinical onset of BSE-H in intracranially inoculated cattle by Konold and colleagues [40]. Based on numerous clinical observations they estimated clinical onset of signs in 4 animals to be 10, 14, 16 and 16 months post inoculation (MPI), with incubation times of 17, 21, 18 and 19 months respectively [40,41]. Our objective and non-invasive measures of retinal function and retinal thickness would have correctly identified 94.7% and 88.9% of all inoculated cattle at 12 MPI respectively, though incubation times ranged from 16.8 months to 31 months.

Our previous work has also demonstrated that changes in retinal function are detectable months prior to other signs of disease in cattle infected with transmissible mink encephalopathy [24]. Similarly, results presented here demonstrate that changes in retinal function as demonstrated by electroretinogram, specifically prolongation of the b-wave implicit time, are detectable at least 5 months prior to when cattle developed unequivocal signs of disease and were euthanized. A significantly prolonged b-wave implicit time, without a significant decrease in b-wave amplitude is rarely observed, and only in individuals that have a mutation that effects synaptic transmission in the outer plexiform layer [42,43]. Despite their significant difference in incubation time, inoculation with both BSE-H and classical BSE resulted in a prolonged b-wave implicit time at 12 months post inoculation. However, the magnitude of the b-wave implicit time prolongation was greater in BSE-H, which had a shorter incubation period. In addition, though b-wave amplitude was not significantly decreased in animals of either group, the b-wave amplitude was notably decreased in BSE-H inoculated cattle at the time of onset of clinical disease. Decreased b-wave amplitude in individuals clinically affected with prion disease has been previously reported in sheep [22,23] and humans [44,45]. Thus, while decreases in the b-wave amplitude appear to accompany clinical disease, our data suggest that b-wave implicit time is a much more sensitive measure of preclinical changes in the retina associated with incubation of a TSE.

Though there were only subtle histopathologic differences between retinas from cattle inoculated with classical BSE and BSE-H, immunohistochemical analysis did demonstrate differences in accumulation of PrP^Sc^ and activation of Müller glia and microglia, with BSE-H having markedly more PrP^Sc^ accumulation, Müller glia and microglia activation. Interestingly, these pathologic features correlate with the more robust phenotype of the electroretinograms in clinically ill BSE-H cattle. That is, when comparing clinical values from BSE-H to classical BSE inoculated animals, b-wave implicit times are longer and their amplitudes are markedly smaller. The b-wave amplitude can be used as an indirect measure of photoreceptor function, as death of photoreceptors results in a decrease in the b-wave amplitude. Though cattle inoculated with BSE-H did have a decreased b-wave amplitude and animals inoculated with classical BSE did not, we did not observe any major differences in the photoreceptor layer between these two groups. This may be due to changes in photoreceptor number in BSE-H cattle below our level of detection, or instead impairment of synaptic communication between photoreceptors and their post-synaptic cells (the bipolar cells). The b-wave implicit time (time for peak to reach maximum amplitude) is generated by retinal bipolar cells and Müller glia [39]. Of these two cell types, we observed changes in only the Müller glia. Both the decrease in b-wave amplitude and the increase in b-wave implicit time can be explained by synaptic dysfunction in the outer plexiform layer, the synaptic interface between photoreceptors and bipolar cells. This would be consistent with accumulation of PrP^Sc^ causing synaptic dysfunction (reviewed in [46]), and correlates with the differences in PrP^Sc^ accumulation between classical BSE and BSE-H.

The more robust retinal histologic changes in BSE-H inoculated cattle also correlates with a shorter incubation time in these animals. It has been shown previously that PrP^Sc^ from BSE-H has a higher stability when compared to classical BSE [47]. Our observation that the higher stability BSE-H has a shorter incubation time, and accumulates in the soma of retinal ganglion cells is consistent with similar observations by Ayers et al, who reported shorter incubation times and intraneuronal accumulation of PrP^Sc^ in higher stability prion strains [48]. In addition, we observed more a robust activation of retinal Müller glia and microglia in retinas from animals inoculated with BSE-H. Experiments to study the relationship between PrP^Sc^ stability, incubation time and glial activation are ongoing.

As an extension of the central nervous system, the retina may have diagnostic potential for several protein misfolding neurodegenerative disorders. Recent studies demonstrate that both retinal function and morphology in patients with Parkinson’s disease are significant predictors of disease severity and quality of life [49–53]. Changes in the retinal nerve fiber layer measured by OCT have been reported in patients with Alzheimer’s Disease [54], and measurements of the choroid (the vascular rich layer of the eye deep to the retina) have shown significant choroidal thinning in AD patients as well [55].

Here we demonstrate that the retina is an important tool to study the pathogenesis of prion disease. The retina is an isolated structure, and thus accumulation of PrP^Sc^ can be precisely quantified, as compared to the rest of the brain where quantification of regional accumulation is affected by dissection. Further, different functional assessment approaches can test different cell populations. The assessments used in this work test photoreceptors, bipolar cells and Müller glia, and our results demonstrate that our functional assessment was sensitive enough to differentiate between classical BSE and BSE-H, which at terminal stages have differences in PrP^Sc^ accumulation, Müller glia and microglia activation However, the results presented here are from animals inoculated with BSE intracranially. Ongoing studies will determine if similar changes can be detected in animals inoculated by the oronasal route.

The suitability of retinal assessment for *diagnosis* of prion disease in animals or humans remains an open question. Unequivocal diagnosis of prion disease depends upon the detection of misfolded prion protein (PrP^Sc^). Several promising diagnostics for Creutzfeldt-Jakob Disease (CJD; the most common human prion disease) include amplification of PrP^Sc^ from nasal brushings [16], blood [15] and urine [14]. Detection of PrP^Sc^ associated with nasal brushings appears to be highly sensitive and specific and can detect both sporadic CJD as well has genetic CJD [16]. Experimentally, PrP^Sc^ can be detected prior to clinical illness in blood from macaques infected with BSE (the agent of variant CJD), and in blood samples from a small number of human CJD patients. PrP^Sc^ was detectable in patients with variant CJD and not sporadic CJD [15], raising the possibility that PrP^Sc^ in blood may be specific to vCJD. In a separate study of human vCJD patients, PrP^Sc^ was detected in the urine from 13/14 individuals [14,15]. The latter two studies demonstrate the utility of bodily fluids for detection of vCJD in clinical and potentially pre-clinical individuals. Though the retinal changes that we describe precede the clinical phase of illness in cattle, it is not yet known how this may relate clinical disease in humans. However, our results, taken with retinal imaging studies of individuals with Parkinson’s and Alzheimer’s disease, suggest that retinal imaging of CJD patients may prove useful.

The preponderance of evidence demonstrates that the retina is affected by protein misfolding disorders long thought to be confined to the brain. Thus, the retina holds tremendous potential for the study of disease pathogenesis, and evaluation of potential therapeutic interventions for multiple protein misfolding disorders. Transmissible spongiform encephalopathies are an infectious and highly predictable model of protein misfolding neurodegenerative disease. Predictable incubation times and PrP^Sc^ accumulation paired with detectable preclinical morphologic and functional deficits make the retina an excellent model for future studies to understand the detailed relationship between accumulation of misfolded protein and specific changes in neural function.

## Supporting Information

S1 Table Summary of Individual Animal Data. Data for each animal includes baseline and 12 MPI values for ERG and OCT, incubation time and indel 12 and indel 23 genotype. From the baseline and 12MPI data 95% CI was calculated and applied to each individual to estimate a specificity and sensitivity for this assay to detect BSE inoculated animals at 12 MPI.
(PDF)

S1 Dataset Individual Animal Electroretinography Data. For each animal in the study, raw values for B-wave amplitude (μV) and B-wave implicit time (miliseconds) are given for each time point data was recorded from that animal. Animals with a red tab were inoculated with classical BSE and animals with a green tab were inoculated with US BSE-H.
(XLSX)

S2 Dataset Individual Animal Optical Coherence Tomography Data. For each animal in the study, raw values for retinal thickness (μM) are given for each time point data was recorded from that animal. Animals with a red tab were inoculated with classical BSE and animals with a green tab were inoculated with US BSE-H.
(XLSX)
