# Supplementary material for: Changes in Retinal Function and Morphology Are Early Clinical Signs of Disease in Cattle with Bovine Spongiform Encephalopathy
Source: PLoS One. 2015 Mar 10;10(3):e0119431. doi: 10.1371/journal.pone.0119431 (PMC4355414; doi:10.1371/journal.pone.0119431)
Supplement: S1 Table — Data for each animal includes baseline and 12 MPI values for ERG and OCT, incubation time and indel 12 and indel 23 genotype. From the baseline and 12MPI data 95% CI was calculated and applied to each individual to estimate a specificity and sensitivity for this assay to detect BSE inoculated animals at 12 MPI. (PDF) [file pone.0119431.s001.pdf]

Classical	-20 dB (milisec) Baseline	-20 dB (milisec) 12 MPI	0 dB (milisec) Baseline	0 dB (milisec) 12 MPI	OCT (uM) Baseline	OCT (uM) 12 MPI	Incubation Time (Months)	23 bp indel	12 bp indel
6825	37.3	59.2	25.4	30.5			21.0	+/-	+/-
6826	54.8	76.8	24.8	35.8	255		17.8	-/-	-/-
6830	63.3	49.4	35.8	31.8	306	241	31.3	+/-	+/-
6836	59.5	67.3	25.2	39.5			21.3	+/+	+/+
6847	57.5	61.3	31.3	25.3			22.9	-/-	-/-
6850	59.7	61	32.4	37.0			31.3	-/-	-/-
6862	56.8	65.8	25.6	34.3			17.3	+/+	+/+
6865	52.0	63.2	29.8	32.3			29.5	+/+	+/+
6870	53.3	61.8	34.5	49.0	303	232	17.6	+/-	+/-
6877	55.7	66.7	24.5	40.7			23.8	+/-	+/-
6992	54.2	63	24.3	30.5			21.3	+/+	+/+
6864					303	246	17.9	-/-	-/-
Means	54.9	63.2	28.5	35.1	288.4	243.8			

BSE-H	-20 dB (milisec) Baseline	-20 dB (milisec) 12 MPI	0 dB (milisec) Baseline	0 dB (milisec) 12 MPI	OCT (uM) Baseline	OCT (uM) 12 MPI	Incubation Time (Months)	23 bp indel	12 bp indel
6873	52.3	65.5	28.3	39			17.7	-/-	-/-
6874	59.6		29.3				16.8	+/-	+/-
6880	59.1	87	27.9	56.7	326.0	264.7	16.3	-/-	-/-
6881	49.8		25.0		260.8	230.6	17.0	-/-	-/-
6911	50.33	60.8	24.5	34.8	317.7	265.8	17.0	+/+	+/+
6912	49.4	72.5	23.7	32.5			17.0	+/-	+/-
6913	50.3		21.9				16.8	+/-	+/-
6973	54.0	68.3	23.5	44.8	328.8	321.5	17.7	+/+	+/+
7012	47.2	73.7	24.3	42.2	320.3	280.2	17.7	-/-	-/-
Mean	52.7	55.38	25.4	41.7	302.2	272.8			

	-20dB Baseline	-20dB 12 MPI	0dB Baseline	0dB 12 MPI	OCT Baseline	OCT 12 MPI	
Control Animals					302.		
					290.		
					290.		
					295.		
					320.		
					286.		
					294.		
					268.		
Pooled Means	53.99 (1.3)	66.8 (2.0)	27.1 (0.9)	37.5 (1.9)	303.4 (5.3)	259.9 (9.5)	
95% CI		61.8-70.3		33.5-41.4		238-282	
False positives	1/19		2/20		2/13		
Specificity	94.7%		90%		84.6%		
Positive positives		18/19		16/17		8/9	
Sensitivity		94.7%		94.1%		88.9%	

Supplementary Table 1: Summary of Individual Animal Data. Data for each animal includes baseline and 12 MPI values for ERG and OCT, incubation time and indel 12 and indel 23 genotype. Standard error for pooled means is in parenthesis. From the baseline and 12MPI data 95% CI was calculated and applied to each individual to estimate a specificity and sensitivity for this assay to detect BSE inoculated animals at 12 MPI.
